# Application of machine learning for lung cancer survival prognostication—A systematic review and meta-analysis

**DOI:** 10.3389/frai.2024.1365777

**Published:** 2024-04-05

**Authors:** Alexander J. Didier, Anthony Nigro, Zaid Noori, Mohamed A. Omballi, Scott M. Pappada, Danae M. Hamouda

**Affiliations:** ^1^Department of Medicine, University of Toledo College of Medicine and Life Sciences, Toledo, OH, United States; ^2^Division of Pulmonary and Critical Care Medicine, Department of Medicine, University of Toledo College of Medicine and Life Sciences, Toledo, OH, United States; ^3^Department of Anesthesiology, The University of Toledo College of Medicine and Life Sciences, Toledo, OH, United States; ^4^Division of Hematology and Oncology, Department of Medicine, The University of Toledo College of Medicine and Life Sciences, Toledo, OH, United States

**Keywords:** artificial intelligence, machine learning, lung cancer, prediction model, algorithm

## Abstract

**Introduction:**

Machine learning (ML) techniques have gained increasing attention in the field of healthcare, including predicting outcomes in patients with lung cancer. ML has the potential to enhance prognostication in lung cancer patients and improve clinical decision-making. In this systematic review and meta-analysis, we aimed to evaluate the performance of ML models compared to logistic regression (LR) models in predicting overall survival in patients with lung cancer.

**Methods:**

We followed the Preferred Reporting Items for Systematic Reviews and Meta-Analysis (PRISMA) statement. A comprehensive search was conducted in Medline, Embase, and Cochrane databases using a predefined search query. Two independent reviewers screened abstracts and conflicts were resolved by a third reviewer. Inclusion and exclusion criteria were applied to select eligible studies. Risk of bias assessment was performed using predefined criteria. Data extraction was conducted using the Critical Appraisal and Data Extraction for Systematic Reviews of Prediction Modeling Studies (CHARMS) checklist. Meta-analytic analysis was performed to compare the discriminative ability of ML and LR models.

**Results:**

The literature search resulted in 3,635 studies, and 12 studies with a total of 211,068 patients were included in the analysis. Six studies reported confidence intervals and were included in the meta-analysis. The performance of ML models varied across studies, with C-statistics ranging from 0.60 to 0.85. The pooled analysis showed that ML models had higher discriminative ability compared to LR models, with a weighted average C-statistic of 0.78 for ML models compared to 0.70 for LR models.

**Conclusion:**

Machine learning models show promise in predicting overall survival in patients with lung cancer, with superior discriminative ability compared to logistic regression models. However, further validation and standardization of ML models are needed before their widespread implementation in clinical practice. Future research should focus on addressing the limitations of the current literature, such as potential bias and heterogeneity among studies, to improve the accuracy and generalizability of ML models for predicting outcomes in patients with lung cancer. Further research and development of ML models in this field may lead to improved patient outcomes and personalized treatment strategies.

## Introduction

### Lung cancer: a significant health challenge

Lung cancer is the leading cause of cancer-related mortality in the United States, with an estimated 225,000 new cases diagnosed annually and 160,000 deaths (Miller et al., 2016). While advances in treatment have led to decreasing trends in mortality and improved survival over the past decade, the median survival remains a dismal 14 months (Howlader et al., 2020; Hu et al., 2021). Lung cancer is broadly classified into two subtypes: non-small cell lung cancer (NSCLC) and small-cell lung cancer (SCLC). NSCLC, the most common subtype, has a 5-year relative survival rate of just 22.9% (National Cancer Institute: Surveillance, Epidemiology, End Results Program, 2022). This is largely due to the fact that most patients are diagnosed at advanced stages, rendering curative treatment options such as surgery ineffective. For these patients, the development of a system to accurately predict survival could aid in treatment and management decisions.

### Current state of lung cancer survival prediction models

The majority of models developed for survival prediction are based on logistic regression (LR), which models the probability of an event occurring based on a linear combination of one or more independent variables. Logistic regression relies on the operator's input, meaning that the programmer must recognize the potential interactions occurring between datapoints in order to develop an accurate model. To overcome this limitation, focus has shifted toward machine learning (ML). Machine learning, a subset of artificial intelligence, is a rapidly growing field that may begin to serve an important function in assisting physicians and patients (Rajkomar et al., 2019). ML algorithms develop a model based on a sample of data (training data) in order to make predictions using mathematical and statistical approaches. Deep learning (DL), a further subset of machine learning, is based on artificial neural networks that mimic neurons in the human brain. These neurons can interact with one another and detect patterns in large datasets without relying on human interaction, allowing them to make accurate predictions. Interest in these techniques has grown as they continue to demonstrate promise in different applications, including survival prediction, treatment recommendations, and image classification. Similarly, a number of image classification methods have been developed for detection of diseases such as COVID-19 and pneumonia using chest radiographs, with strong results that may be applicable to lung cancer survival models (Zumpano et al., 2021; Rani et al., 2022a). Contributions focused on chest radiograph preprocessing techniques and techniques enabling 3D visualization have allowed for denoising of images, leading to heightened prediction accuracy (Rani et al., 2022b; Pradhan et al., 2023). Advancements in technology have allowed for the development of survival prediction models that may assist clinicians to make personalized decisions for their patients on aspects such as follow-up timeline or supportive care roles. The downstream effects of these models could significantly reduce physician burnout and improve the efficiency of our healthcare system. However, recent analyses have challenged the notion that machine learning models may be superior to those developed using logistic regression (Christodoulou et al., 2019; Sufriyana et al., 2020). ML models hold significant potential to improve healthcare spending and decision making for physicians and patients, however, their performance in predicting lung cancer outcomes has been largely underexplored in the current literature.

### Rationale

In this study, we systematically review the current state of the literature surrounding the development and use of machine learning models in predicting survival in patients with lung cancer. Further, we employ meta-analytic estimates to compare the accuracy of machine learning algorithms with those of traditional logistic regression models in predicting survival of patients with lung cancer.

## Materials and methods

We followed the Preferred Reporting Items for Systematic Reviews and Meta Analysis (PRISMA) statement (Moher et al., 2009). The PRISMA steps that were followed include developing eligibility criteria, then selecting information sources, creating a search strategy, selecting studies that meet eligibility criteria, defining variables and data extracted from each study, then assessing risk of bias in individual studies.

### Search strategy

A search query was designed based on a previously published study evaluating machine learning in cardiac surgery (Benedetto et al., 2022). We modified the original query by adapting the keywords to select relevant studies for lung cancer, as opposed to cardiac surgery. Keywords were selected to capture results from lung cancer, artificial intelligence, and outcomes data. These keywords were strung together using Boolean operators to develop the following novel query: “*lung cancer*” AND (*outcomes* OR *risk* OR *prediction* OR *mortality* OR *prognosis* OR *survival*) AND (*machine learning* OR *artificial intelligence* OR *deep learning* OR *neural network* OR *random forest* OR *decision tree* OR *support vector machine*). These machine learning algorithms were included in the search query due to their use in studies evaluating the accuracy of machine learning for survival prediction (Kourou et al., 2015). This search query was inputted into the Medline (2,940), Embase (6,593), and Cochrane (2) databases on 06/01/2023. All abstracts were independently screened by two reviewers (AD and AN). Disagreements in study selection were resolved by a third, experienced reviewer.

### Inclusion and exclusion criteria

Studies were eligible for inclusion if they met the following criteria: (1) originally written in the English language; (2) article described the development of an ML model to predict overall survival in patients aged ≥18 with lung cancer and compared the performance of the ML model with an LR model using the same dataset. Studies were excluded if they were not written in English, did not use an ML model, did not study patients aged ≥18 with lung cancer, did not predict overall survival, or did not compare an ML model with LR using the same dataset. Review articles, case reports, conference proceedings, editorials, abstract-only articles or articles without full text were excluded. Papers from conference proceedings were excluded due to the potential for preliminary findings that have not undergone extensive validation or peer review, in addition to a potential for limited details on methods, results, and conclusions.

### Risk of bias assessment

We followed the methods described by Christodoulou et al. (2019) to evaluate risk of bias in studies of ML algorithms. We defined five signaling items to indicate potential bias: (1) unclear or biased validation of model performance; (2) difference in whether data-driven variable selection was performed before applying LR and ML algorithms; (3) different predictors considered for LR and ML algorithms; (4) whether corrections for imbalanced outcomes were used only for LR or only for ML algorithms; and (5) difference in handling of continuous variables before applying LR or ML algorithm. Each bias item was scored as no (not present), unclear, or yes (present). We considered a comparison at low risk of bias if the answer was “no” for all five signaling items. If the answer was “unclear” or “yes” for at least one item, we assumed high risk of bias.

### Data extraction

Two reviewers (AD and AN) extracted data from each study. The extracted items were based on the Critical Appraisal and Data Extraction for Systematic Reviews of Prediction Modeling Studies (CHARMS) checklist and the Quality Assessment for Diagnostic Accuracy Studies-Comparative (QUADAS) risk of bias tool (Whiting et al., 2011; Moons et al., 2014). The CHARMS checklist was developed using methodological recommendations for data extraction, risk-of-bias tools, and data extraction protocols from previously published systematic reviews of prediction modeling studies to guide collection of data for systematic reviews. This checklist represents an unbiased method of data extraction for systematic reviews. Items included in the checklist include source of data, predicted outcomes, sample sizes, handling of missing data, model development, model performance, model evaluation, and results. Additionally, we extracted the year the study and the first author's affiliated country to assess for potential geographic bias.

### Meta-analytic analysis

Our primary objective was to compare the discriminative ability for overall survival of ML models with that of LR models. Discriminative ability refers to the capacity of a model to accurately predict whether an event will or will not happen, as measured by the concordance index (C-statistic). The C-statistic corresponds with the area under the receiver operating characteristic curve (AUC), which plots sensitivity (true positive) against 1 – specificity (false positive), to demonstrate the relationship between these two variables and represents a measure of clinical utility. A C-statistic of 0.5 corresponds with random, or a 50% chance, of an event occurring, whereas a C-statistic of 1.0 indicates a model has 100% discriminative ability.

After extraction, the C-statistics for the highest performing models in both groups were summarized into a weighted average. Pooled C-statistics were compared using a previously described method. Sensitivity analysis was conducted using the one-removal function (Lee, 2018). In this analysis, each study included in the meta-analysis is systematically excluded one at a time, and the meta-analysis is rerun to determine how the exclusion of each study affects the pooled effect size or outcome. We elected not to utilize a funnel plot due to a small sample size of studies included in the meta-analytic estimates. Models must have reported a 95% confidence interval to be included in the pooled analysis. All tests were two-tailed and *p*-values ≤ 0.05 were considered statistically significant. A random effects model was used to determine the meta-analytic estimates. All statistical analysis was performed using RStudio version 2022.02.3.

## Results

### Literature search

**Figure 1:**
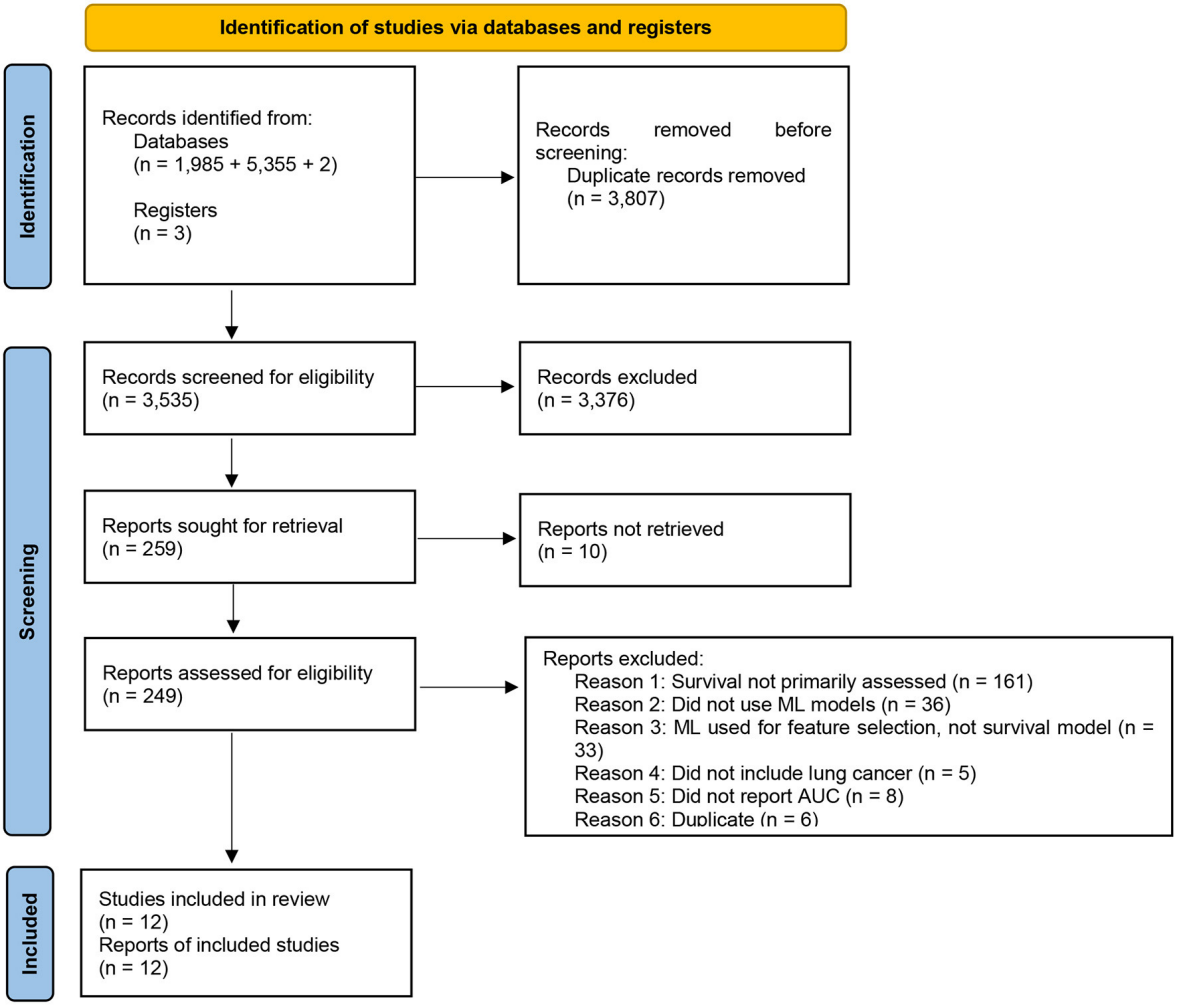
PRISMA flow diagram of screening process.

The search and screening of studies are demonstrated in Figure 1. Our search resulted in 3,635 studies published between 2006 and 2022. We included 259 studies based on title and abstract. After full-text screening, we excluded 247 studies and 12 studies met inclusion criteria and qualitative and quantitative data was extracted. Of these 12, we included six which reported confidence intervals in our meta-analysis. None of the studies had an overlapping population.

### Study characteristics

Twelve studies were analyzed, with 23 datasets and 211,068 patients eligible for inclusion in the systematic review, including six studies in the meta-analysis (Bartfay et al., 2006; Elfiky et al., 2018; Jochems et al., 2018; Siah et al., 2019; Afshar et al., 2020; Huang et al., 2020; Wang et al., 2020; Loureiro et al., 2021; Chan et al., 2022; Hindocha et al., 2022; Yang et al., 2022; Zhang et al., 2022) (Tables 1, 2). All of the studies were retrospective analyses. Most of the studies were published in 2018 or beyond (*n* = 11, 92%). 5 of the studies were published in China (42%) and 3 (25%) in the United States. All but one of the studies included clinical predictors in their model, with one study using only radiological data to predict survival outcomes. The median number of predictors used to build the machine learning model was 17, which ranged from 6 to 5,390. The most common model deployed was random forest (*n* = 4, 33%) followed by artificial neural network (*n* = 3, 25%). Six studies demonstrated random forest as their best performing model (50%), while the following 5 (42%) listed XGboost as their best performing model and one study listed an ensemble model as their best performing. Nine studies described machine learning models which outperformed logistic regression by a mean difference of 9%.

**Table 1:** Characteristics of the studies included in the systematic review.

**References**	**Country**	**Patient Pop**.	**Predictors**	**Sample (*n*)**	**ML AUC**	**LR AUC**
Afshar et al. (2020)	Canada	Early-stage lung cancer	Radiomics Clinical	132	0.68	0.51
Hindocha et al. (2022)	UK	Stage I–III NSCLC	Clinical Radiomic	657	0.717	0.665
Jochems et al. (2018) and Huang et al. (2020)	China	All NSCLC with bone mets	Clinical	6,087	0.786	0.751
Yang et al. (2022)	China	Stage III NSCLC	Clinical Histological	16,781	0.665	0.629
Jochems et al. (2018)	Netherlands	All NSCLC	Clinical	1,005	0.66	0.55
Loureiro et al. (2021)	Germany	Advanced NSCLC	Clinical	137,906	0.665	0.671
Chan et al. (2022)	China	All NSCLC	Radiomics	123	0.675	0.765
Bartfay et al. (2006)	Canada	SCLC	Clinical	608	0.654	0.645
Elfiky et al. (2018)	USA	All lung cancer	Clinical	28,873	0.771	0.537
Zhang et al. (2022)	China	All lung cancer	Clinical Radiomic	420	0.66	0.57
Siah et al. (2019)	USA	Advanced NSCLC	Clinical	7,805	0.725	0.726
Wang et al. (2020)	China	IB-IIA stage NSCLC.	Clinical	10,671	0.6367	0.5612

**Table 2:** Selected contributions of the studies examined.

**References**	**Source of data**	**Type of model**	**Best performing model**
Afshar et al. (2020)	Institutional registry	CNN (DL)	ANN (CNN)
Hindocha et al. (2022)	3 UK NHS Registries	Ensemble of MDA XGB NNET	Ensemble
Huang et al. (2020)	SEER database Institutional registry	RF SVM	RF (XGB)
Yang et al. (2022)	SEER database Institutional registry	ANN	ANN
Jochems et al. (2018)	4 Institutional registries Maastro database	RF	RF
Loureiro et al. (2021)	Flatiron Health Database OAK clinical trial database	Regularized Cox, RSF, Gradient Boosting (GB), DeepSurv (DS), Autoencoder (AE), Super Learner (SL)	RF (RSF)
Chan et al. (2022)	Institutional registry	RF	RF (XGB)
Bartfay et al. (2006)	National cancer registry	ANN	ANN
Elfiky et al. (2018)	Institutional registry	RF	RF (XGB)
Zhang et al. (2022)	Institutional registry	ANN	ANN (CNN)
Siah et al. (2019)	17 randomized clinical trials	RF	RF (RSF)
Wang et al. (2020)	West China Hospital	ANN	ANN

85.8% (*n* = 181,035) of the patients included had NSCLC, 13.9% were unreported, and 0.2% (*n* = 608) had SCLC. 71.9% (*n* = 151,798) patients had advanced stage (Stage IV) lung cancer at diagnosis, while 13.4% (*n* = 28,241) patients had early stage (I–III) lung cancer and 14.4% (*n* = 30,421) were unknown.

### Methodological quality

The risk of bias assessments for each analyzed study are shown in Table 3. Of the 12 studies included in the systematic review, nine had low risk of bias. The three studies which were identified as high risk of bias all had differences in the variables selected as predictors between the machine learning and logistic regression models, which led to a high risk of bias. Additionally, the study by Jochems et al. included an unclear validation cohort.

**Table 3:** Assessment of bias of the studies included in the systematic review.

**Study information**	**Bias assessment**					**Risk of bias**
**References**	**Unclear or biased validation of performance**	**Difference in use of data-driven variable selection**	**Difference in handling of continuous variables**	**Difference in considered predictors**	**Difference in methods for class imbalance**	
Afshar et al. (2020)	Low	Low	Low	Low	Low	Low
Hindocha et al. (2022)	Low	Low	Low	High	Low	High
Huang et al. (2022)	Low	Low	Low	Low	Low	Low
Yang et al. (2022)	Low	Low	Low	Low	Low	Low
Jochems et al. (2018)	Unclear	Low	Low	High	Low	High
Loureiro et al. (2021)	Low	Low	Low	Low	Low	Low
Wang et al. (2020)	Low	Low	Low	Low	Low	Low
Bartfay et al. (2006)	Low	Low	Low	Low	Low	Low
Elfiky et al. (2018)	Low	Low	Low	Low	Low	Low
Zhang et al. (2022)	Unclear	Low	Low	High	Low	High
Siah et al. (2019)	Low	Low	Low	Low	Low	Low
Wang et al. (2020)	Low	Low	Low	Low	Low	Low

### Meta-analytic estimates

**Figure 2:**
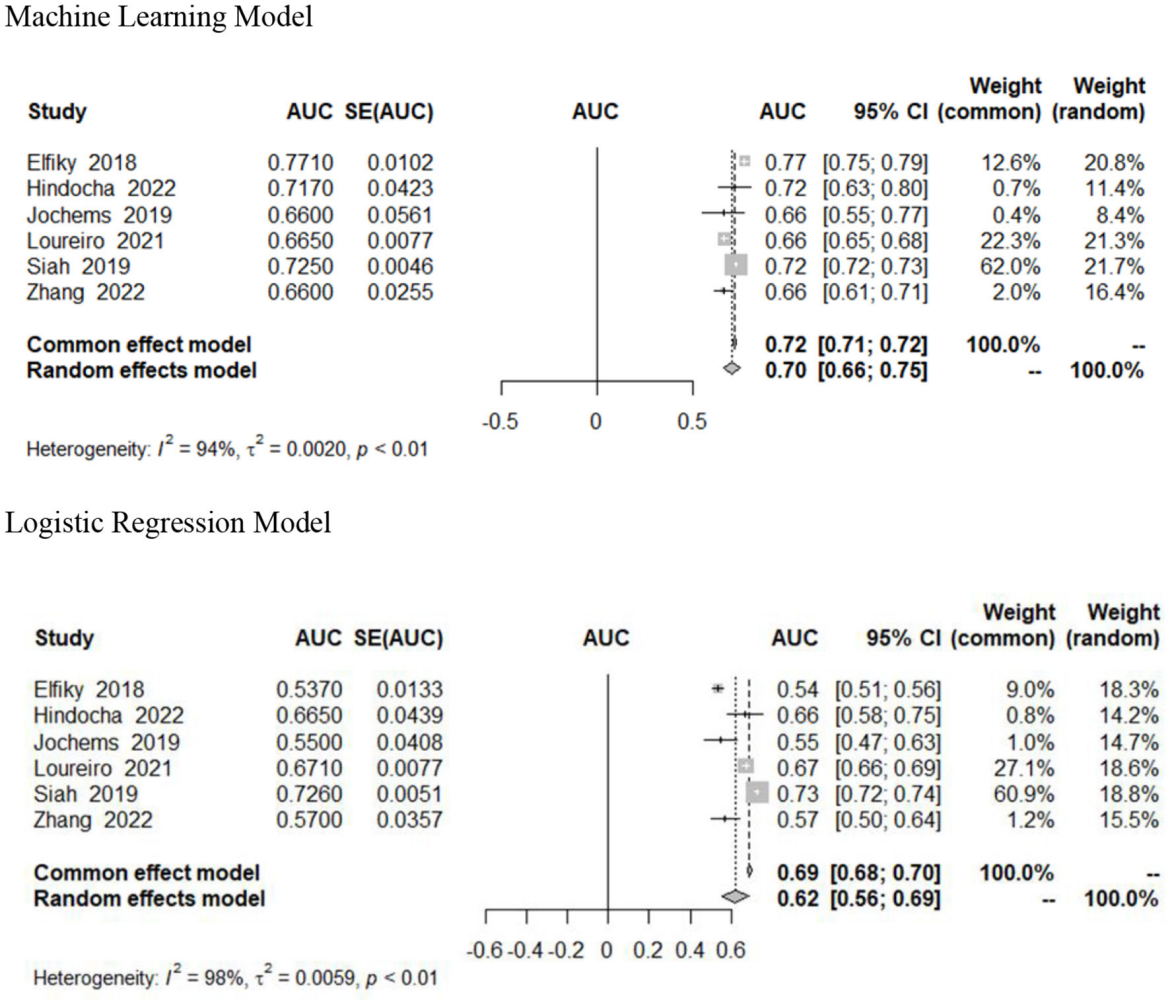
Forest plot of ML and LR AUC.

Six studies included 95% confidence intervals in their estimates and were included in our pooled analysis measuring the AUC of survival prediction models (Figure 2) (Elfiky et al., 2018; Jochems et al., 2018; Siah et al., 2019; Loureiro et al., 2021; Hindocha et al., 2022; Zhang et al., 2022). The AUC ranged from 0.66 to 0.77. The random effects estimate was 0.70 (95% CI 0.66–0.75) for machine learning models and 0.62 (95% CI 0.56–0.69) for logistic regression models. Machine learning models outperformed traditional (or without use of ML) logistic regression models; however, this was not statistically significant (Table 2). There was significant heterogeneity (*I*^2^ = 94%, *p* < 0.01). We assessed heterogeneity using the One-study removed function, which did not reveal any decrease in heterogeneity after removal of studies.

## Discussion

To the authors' best knowledge, this is the first study to perform both a systematic review and a meta-analysis of the accuracy of machine-learning based models at predicting survival outcomes of patients with lung cancer. Our meta-analytic results demonstrate that machine-learning based models have modest proficiency at predicting survival in this patient population. These capabilities are strong but did not outperform their logistic regression counterparts in our analysis.

### Model performance in our study

The best performing model in our sample was the XGBoost algorithm developed by Huang et al. (2020). They used clinical datapoints, including race, age, sex, marital status, tumor site and size, TNM staging (a cancer staging system using tumor characteristics such as size, number of lymph nodes positive for malignancy, and presence of metastasis), treatment modalities received, and histological type and grade to predict the 1-year overall survival of patients with NSCLC who had bone metastases. Using the SEER database for training and an institutional dataset for validation, their model achieved an AUC of 0.786. The XGBoost model is a decision tree system that represents complex relationships between variables. These algorithms are interpretable and transparent, addressing the black-box concerns that surround machine learning models (Holzinger et al., 2017). Additionally, they do not require the sizeable computational resources that other machine learning models require, rendering them more deployable at the individual patient level. For these reasons, the XGboost models have been suggested for larger use within the healthcare system to predict clinical outcomes (Zabihi et al., 2019; Bolourani et al., 2021). This is further reinforced by our study results−42% of studies included in our systematic review demonstrated XGboost models as their highest performing.

### Variables used in development of lung cancer survival models

At the present time, a number of lung cancer prediction models have been developed with the potential to be deployed for use in the healthcare setting. Most of these models incorporate the TNM staging system, which is especially important given the significant prognostic power that factors such as metastasis weigh into survival (She et al., 2020). She et al. (2020). used the SEER dataset to access the listings of 12,912 patients with NSCLC, which served as their training set. They developed a deep learning neural network model for survival prediction using 127 clinical variables, which outperformed their regression-based algorithm that used only TNM staging (C-statistic 0.74 vs. 0.69). However, their results may have been limited by the use of only clinical variables to train the model. Hsu et al. (2022) used an institutional dataset and variables, including demographics, comorbidities, medications, laboratory values, and genetic test results, to develop nine models to predict NSCLC survival. Their best performing model, an artificial neural network, achieved an AUC of 0.89. Notably, of the nine models developed, the traditional LR model exhibited the lowest AUC (and thus, the lowest predictive ability). Lai et al. (2020) developed a deep neural network using integrated microarray data with clinical variables, obtaining a strong predictive performance (AUC = 0.82). Combing the results of multiple dimensions of variables (laboratory testing, genomic testing, imaging, clinical variables, etc) may yield the greatest predictive power due the incorporation of multiple aspects of the clinical picture into the survival prediction.

### Advantages and disadvantages of ML models over traditional survival prediction models

Machine learning models have a number of advantages over traditional logistic regression models. Healthcare is beginning to move toward large sets of data, and the widespread use of the electronic health record enables the creation of large databases such as the National Cancer Database or SEER Database. However, health record data is notably complicated and voluminous. Machine learning models are aptly suited for detecting complex patterns in population-level databases, since they don't necessarily require an operator's manual input and oversight (LeCun et al., 2015). Thus, although the developer may be unable to recognize and specify each interaction between variables used to train the model, the model can recognize and learn from the nonlinear relationships within the data to arrive at a prediction. Further, with new data, the model may be continuously improved (Silver, 2011). This is termed incremental learning and confers a unique advantage to machine learning algorithms when compared with logistic regression.

Despite their advantages, machine learning models also have a number of drawbacks. Although we discussed previously that they can detect patterns in large datasets, this data must be meticulously processed and readable for the machine. This requires accurate labeling of the data used to train the model—any inaccuracy will affect the prognostic impact of the machine. This data can take many forms, from typed physician's notes in the electronic health record to clinical images to histological slides from the pathologist. In light of this heterogeneity, errors in labeling are a consistent source of struggle and require substantial investment of time and effort (van Grinsven et al., 2016). Additionally, the potential bias in the outputs of machine learning models has been a topic of recent discussion (Huang et al., 2022). Racial biases in these models may be especially prevalent (Obermeyer et al., 2019). One study developing clinical models to detect arrythmias was 31% less accurate for Black patients when compared with Asian patients (Alday et al., 2022). These biases are a reflection of the data used to train the models—datapoints demonstrate the systemic inequities present in our healthcare system today, which is physically exhibited by the algorithms. Moving forward, caution must be emphasized in the pipeline of model development so as not to further exacerbate discrepancies in care and outcomes (Chen et al., 2021).

### Use of DL models in lung cancer survival prediction

DL-based approaches such as neural networks are often regarded as “black box” approach due to difficulties investigators may experience in interpreting the data points and variables are deemed (by the model) to be significant predictors of patient outcomes. This is one of the perceived weaknesses in their adoption in medicine despite their better performance with respect to other modeling approaches like LR. ML-based approaches have the ability to capture the relationship between hundreds to thousands of predictors on a specific outcome or output variable of interest. The black-box nature of DL has led to the increasing adoption of hybrid systems that combine ML and DL approaches. These hybrid systems aim to leverage the strengths of deep learning for feature learning while incorporating more interpretable ML models for decision-making. By integrating both approaches, researchers and practitioners can achieve better transparency and understandability while maintaining high predictive performance. Moreover, there is a growing interest in solutions based on argumentation frameworks. Argumentation approaches provide a structured framework for reasoning and decision-making, allowing for the explicit representation of different perspectives, uncertainties, and reasoning paths involved in AI systems (Caroprese et al., 2022). There is a concern that if adopted, healthcare providers may become reliant on the use of such platforms which would result in induction of “automation bias” which may result in unintended incidence of medical error when clinical judgement and expertise is not prioritized. Given that machine learning and AI-based models often lack transparency in both their internal logic and output, it is necessary to develop technologies which promote better transparency in their output and recommendations (Gretton, 2018). While not trivial, improving transparency in machine learning-based technologies maybe feasible via certain methods. Techniques can be implemented to evaluate model weights as they relate to the ultimate model-generated output (Luo et al., 2022). Expanding upon this approach, when the most impactful model input features are identified it is possible to provide a mechanism for users of machine learning-based systems to “simulate” the output of models given user-specified changes in predictors (specified into a software application or decision support platform). Being able to “simulate” the output of a model given some intervention (e.g., medication dosage) or patient characteristic (e.g., weight) defined by a model input or set of model inputs to provide some indication of their relative contribution to a model output of interest. Furthermore, this would improve transparency of model-generated predictions and allude to the potential importance of certain model inputs and their contribution to the ultimate model output. This makes machine learning-based approaches less of a “black box” and provides insight into the input output relationship. As machine learning-based approaches become more commonplace, simulating the impact of different model inputs such as treatment interventions, comorbidities, demographics, etc. and how they influence model predictions is going to be a necessity in the space to improve adoption of machine learning and AI-based systems and technologies.

### Limitations

Our study is not without limitations. We were limited by the small number of studies eligible for inclusion, all of which were retrospective in nature. Three studies in our sample were at high risk for bias, which limits the generalizability of our results. There was no standardized method of feature selection, leading to studies with differing numbers of predictors used and heterogeneity across model development. Regarding our meta-analysis, we were limited by the significant heterogeneity. This heterogeneity may be due to differences in study populations or timing of outcome measurements, as different studies assessed survival at differing time periods. To address this heterogeneity, we used a random-effects model. Further, including various stages of lung cancer may have limited our generalizability. Patients in different stages of their disease may demonstrate different predictors of survival. For example, the location and number and number of metastases in a late-stage patient may represent a sensitive predictor that would not be applicable to a patient with early-stage lung cancer. Due to our small sample size, subgroup analysis across different stages was not possible.

## Conclusion

Machine learning models show promise in predicting overall survival in patients with lung cancer, with superior discriminative ability compared to logistic regression models. However, further validation and standardization of ML models are needed before their widespread implementation in clinical practice. One key challenge is the lack of standardized data collection and integration across different healthcare institutions. Future research should focus on developing robust methods for integrating diverse datasets, including clinical, genomic, and imaging data, to improve the accuracy and generalizability of ML models. Additionally, researchers should focus on addressing the limitations of the current literature, such as potential bias and heterogeneity among studies, to improve the accuracy and generalizability of ML models for predicting outcomes in patients with lung cancer. Additionally, future trends may involve the development of interpretable ML models and techniques for generating transparent explanations of predictions, enhancing trust and acceptance among clinicians and patients. Overall, ML has the potential to enhance prognostication in lung cancer patients and improve clinical decision-making. By addressing these open challenges and embracing potential future trends, the development and implementation of ML models for predicting overall survival in patients with lung cancer can be further advanced, ultimately leading to improved patient outcomes and personalized treatment strategies in clinical practice.

## Data availability statement

The original contributions presented in the study are included in the article/supplementary material, further inquiries can be directed to the corresponding author.

## Author contributions

AD: Conceptualization, Data curation, Formal analysis, Funding acquisition, Investigation, Methodology, Project administration, Resources, Software, Supervision, Validation, Visualization, Writing—original draft, Writing—review & editing. AN: Writing—original draft, Writing—review & editing. ZN: Writing—original draft, Writing—review & editing. MO: Writing—original draft, Writing—review & editing. SP: Writing—original draft, Writing—review & editing. DH: Writing—original draft, Writing—review & editing.
